# Reduced Plasma miR-146a Is a Predictor of Poor Coronary Collateral Circulation in Patients with Coronary Artery Disease

**DOI:** 10.1155/2016/4285942

**Published:** 2016-12-05

**Authors:** Junnan Wang, Youyou Yan, Dandan Song, Bin Liu

**Affiliations:** ^1^Department of Cardiology, Second Hospital of Jilin University, No. 218 Ziqiang Street, Changchun 130041, China; ^2^Department of Clinical Laboratory, Second Hospital of Jilin University, No. 218 Ziqiang Street, Changchun 130041, China

## Abstract

Coronary collateral circulation (CCC), an alternative blood supply for ischemic myocardium, improves survival rates among patients with coronary artery disease (CAD). However, there remains a lack of biomarkers to discriminate between patients with poor or good CCC. In this study, we aimed to observe the relationship between plasma microRNA-146a (miR-146a) levels and the coronary collateral circulation (CCC). Additionally, we aimed to explore whether the plasma miR-146a level could serve as a blood-based biomarker for CCC in patients with CAD. We measured the plasma levels of vascular endothelial growth factor A (VEGF-A) and miR-146a in patients with CCC by ELISA and real-time PCR, respectively, according to the Rentrop grades. The results showed that the plasma miR-146a level is significantly increased in CAD patients with good CCC and significantly decreased in those with poor CCC. In contrast, although VEGFA expression in patients followed a similar trend as the CCC, the differences between the groups were not statistically significant. There was a positive correlation between plasma miR-146a levels and the Rentrop grading. In addition, receiver operator characteristic analysis showed that miR-146a could be a potent biomarker for identifying patients with poor CCC.

## 1. Introduction

Coronary collateral circulation (CCC), an alternative blood supply for ischemic myocardium, improves survival rates among patients with coronary artery disease (CAD) [1]. Identifying patients with poor CCC can, in turn, distinguish patients at risk of substantial debilitation from adverse cardiac events [2]. Currently, evaluation of collateral development depends mainly on invasive procedures such as coronary angiography and grading [3]. Therefore, the identification of a circulating biomarker of CCC status would be of great clinical significance. Although there are many established determinants of coronary angiogenesis, including chronic inflammation and vascular endothelial growth factor (VEGF) [4], there remains a lack of biomarkers to discriminate between patients with poor or good CCC.

Circulating microRNAs (miRNAs)—noncoding RNAs that are ~22 nucleotides long, present in the bodily fluids of patients—are considered to hold potential as novel disease markers [5]. It has been established that miRNAs function as key regulators of different aspects of vascular biology, including angiogenesis [6]. It has been reported that there is a close relationship between angiogenesis and several miRNAs: miR-21, miR-146a, miR-155, miR-221, and miR-222 [7]. All of these have been shown to exert antiangiogenic effects, apart from miR-146a [7]. Although angiogenesis plays an important role in CCC formation [8], the correlation between miR-146a and the CCC has not been substantially investigated.

In this study, we aimed to investigate the correlation of circulating miR-146a with CCC and to test whether miR-146a could serve as a potential biomarker for CCC formation in CAD patients.

## 2. Materials and Methods

### 2.1. Study Population

From December 2015 to July 2016, all patients who underwent coronary angiography at the Second Hospital of Jilin University for Cardiovascular Diseases (Changchun, China) were screened for eligibility, provided that they had at least one major coronary occlusion or a stenosis of ≥ 95%, accompanied by thrombolysis in myocardial infarction- (TIMI-) grade 1 anterograde-flow. A total of 34 consecutive patients with good CCC and 44 patients with poor CCC were retrospectively investigated. The coronary collateral grading was determined according to the Rentrop grades [9, 10]: grade 0, no filling of any collateral vessels; grade 1, filling of side branches of the artery to be perfused by collateral vessels without visualization of epicardial segment; grade 2, partial filling of the epicardial artery by collateral vessels; and grade 3, complete filling of epicardial artery by collateral vessel. Patients with CAD were divided into two groups according to their Rentrop grade: patients with grade 2 or 3 collateral development (good CCC group, *n* = 34), and patients with grade 0 or 1 collateral development (poor CCC group, *n* = 44). In addition, 34 healthy subjects without CAD were enrolled as control subjects. Patients with any of the following factors were excluded from the current study: coronary artery lumen diameter stenosis < 90%, recent history (history of less than one month) of acute coronary syndrome, congestive heart failure, concomitant inflammatory diseases and/or neoplastic diseases, or the use of steroids, immunosuppressive drugs, or nonsteroidal anti-inflammatory drugs (except for low-dose aspirin). Detailed physical examinations and both electrocardiographic and echocardiographic evaluations were performed on all patients. Written consent was obtained from each of the subjects and the study protocol was approved by the Ethics Committee of Jilin University.

### 2.2. Evaluation of Plasma VEGF-A Levels by ELISA

Blood samples (5 mL per patient) were collected from the subjects, via direct venous puncture, into tubes containing sodium citrate, and then centrifuged at 1000 ×g for 5 min; the supernatant (plasma) was then transferred carefully into tubes. We used the “Human VEGF-A ELISA Kit” from Elabscience to measure plasma levels of VEGF-A, according to the manufacturer's instructions. Concentrations of VEGF-A in subject plasma samples were determined by plotting the absorbance of each sample against a standard curve of recombinant human VEGF-A. Absorbance was measured at 450 nm (primary wavelength).

### 2.3. qPCR Assay

MicroRNAs were extracted from the plasma samples using the miRcute miRNA Isolation kit (TRANS GEN, Beijing City, China). The samples were performed both poly-(A) tailing and reverse transcription with the miScript reverse transcription kit (TRANS GEN, Beijing City, China). MiR-146a was quantified by quantitative reverse transcription-polymerase chain reaction (qRT-PCR) assay and U6 RNA was used as the miRNA internal control. Each reaction was carried out using a miRNA specific forward primer and a universal reverse primer according to the protocol of the manufacturer (Tiangen, Beijing City, China). The primers were as follows: miR-146a forward: 5′-TGAGAACTGAATTCCATGGGTT-3′ U6 forward: 5′-GCTTCGGCAGCACATATACTAAAAT-3′. Each reaction was performed in a total volume of 20 *μ*L: 2 *μ*L reverse transcription products, 1.0 *μ*L 20x “Micro RNA assay primer”, 10 *μ*L 2x “Universal PCR Master Mix” and nuclease-free H_2_O to adjust the volume. The PCR reaction was performed as follows: 95°C for 5 min, followed by 40 cycles (95°C for 15 seconds; 60°C for 30 s). Relative gene expression levels were analyzed using the formula 2-ΔCT, where ΔCT = CT (target gene) − CT (control).

### 2.4. Statistical Analyses

*SPSS 16.0* (SPSS Inc., Chicago, IL, USA) was used to perform statistical analyses. Data have been reported as means ± SD, and medians have been calculated for the general characteristics of subjects. Differences among the different groups were assessed using One-Way ANOVA comparisons. *P* values < 0.05 were considered to indicate statistical significance. Relationships between miR-146a, VEGF-A, and collateral grade were assayed using the Spearman correlation test. Receiver operating characteristic (ROC) curves were established to evaluate the predictive power of circulating miR-146a for the CCC status of patients; the area under the ROC curve (AUC) was used to assess the predictive power. Sensitivity and specificity were calculated according to standard formulas.

## 3. Results

### 3.1. Baseline Characteristics

There were 44 patients with poor CCC, 34 with good CCC, and a further 34 healthy control subjects. Their clinical characteristics and biochemical parameters are listed in Table 1. None of the following differed between the groups: age, sex, and/or diabetes mellitus, smoking, and lipid profiles including LDL cholesterol, triglycerides, and total cholesterol. The difference between prevalence of hypertension in poor CCC and control groups was statistically significant (*P* < 0.05).

### 3.2. Levels of Plasma VEGF-A and miR-146a in Patients Classified by Their CCC Status

**Figure 1:**
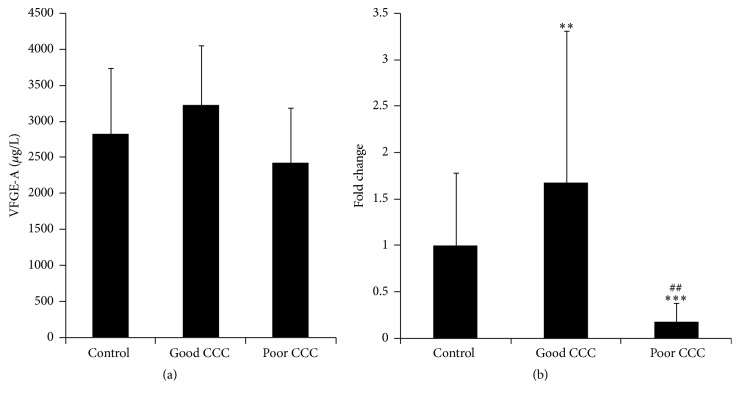
Level of plasma VEGF-A or miR-146a in patients, according to their CCC status. (a) The plasma level of VEGF-A in patients, according to their CCC status. (b) The plasma level of miR-146a in patients, according to their CCC status. Good CCC group versus poor CCC group: ^##^ *P* < 0.01. Poor CCC group versus control group: ^*∗∗∗*^ *P* < 0.01. Good CCC group versus control group: ^*∗∗*^ *P* < 0.05.

It has been reported that miR-146a can exert proangiogenic effects [7] and can upregulate VEGF-A [11], which is one of the most important vascular growth factors during angiogenesis. In this study, we measured levels of plasma VEGF-A and miR-146a in patients that had been classified by their CCC status. The concentrations of plasma VEGF-A in the control, good CCC, and poor CCC groups were 2827.44 ± 914.07 *μ*g/L, 3232.06 ± 820.07 *μ*g/L, and 2425.30 ± 761.57 *μ*g/L, respectively. Relative to that in the control group, the plasma level of VEGF-A was higher in the good CCC group and lower in the poor CCC group; however, these differences were not statistically significant (Figure 1(a)).

We next tested the levels of plasma miR-146a in patients, according to their CCC status. Our results showed that, relative to the control group, the level of plasma miR-146a was higher in patients with good CCC (1.67-fold, *P* < 0.05), whereas it was lower in patients with poor CCC 0.17-fold, (*P* < 0.01). The difference between the level of plasma miR-146a in the good and poor CCC groups was statistically significant (*P* < 0.01) (Figure 1(b)).

### 3.3. Correlation between miR-146a and Rentrop Grades in Patients with Different CCC Status

**Figure 2:**
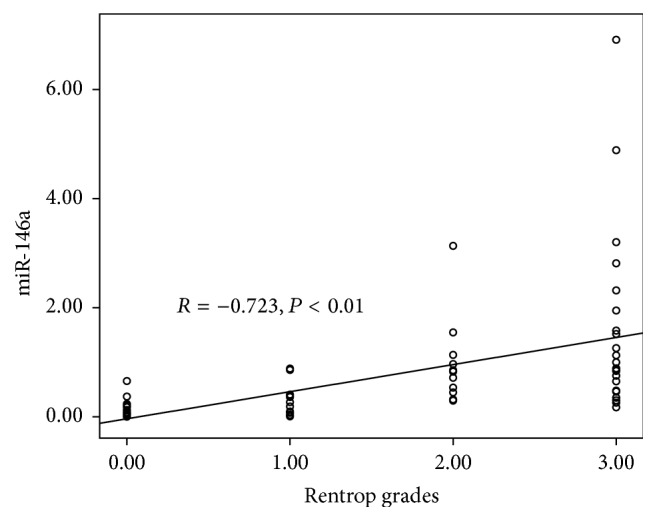
Correlation between the level of plasma miR-146a and the Rentrop grades.

To investigate the relationship between levels of plasma miR-146a and CCC status, we next analyzed the correlation between miR-146a and patient Rentrop grades, classified by their CCC. A positive correlation was identified between miR-146a and the Rentrop grading (*R* = −0.723, *P* < 0.01) (Figure 2). In addition, we also tested the correlation between the levels of miR-146a and VEGF-A, but no statistically significant difference could be identified (data not shown).

### 3.4. Predictive Power of miR-146a for CCC

**Figure 3:**
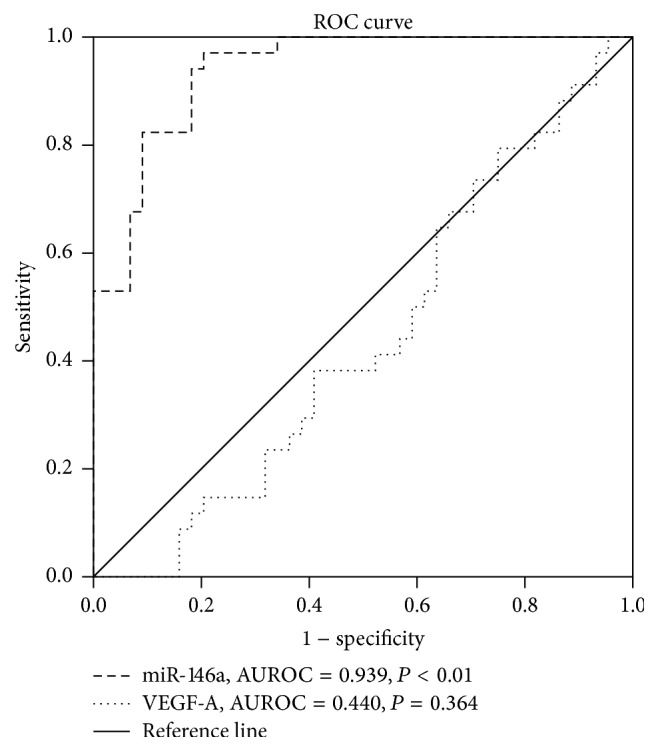
Comparisons of the sensitivity and specificity of levels of plasma miR-146a or VEGF-A for the diagnosis of CCC in patients.

To evaluate the predictive power of the level of circulating miR-146a for a patient's CCC status, we performed ROC analysis on all 78 patients with CCC. As shown in Figure 3, the areas under the ROC curve (AUROC) were 0.939 for miR-146a (95% confidence interval, *P* < 0.01) and 0.440 for VEGF-A (95% confidence interval, *P* = 0.364). Therefore, the level of circulating miR-146a had marked sensitivity and specificity for CCC, and it was superior to VEGF-A for diagnosis.

## 4. Discussion

In this study, we found that levels of plasma miR-146a were significantly lower in the poor CCC group and significantly higher in the good CCC group, as compared with that in the control group. In addition, levels of circulating miR-146a positively correlated with the Rentrop grading of the source patients. Our ROC analysis demonstrates that the level of plasma miR-146a could be used as a potent independent predictor of poor CCC.

It had been found that there is the close relationship between miR-146a and angiogenesis [7]. Our data are consistent with this earlier finding because we discovered that the level of miR-146a is increased in CAD patients with good CCC and decreased in those with poor CCC. Similarly, it had been demonstrated that miR-146a could upregulate VEGF-A [11], a proangiogenic factor. In our study, although the trend of VEGF-A expression in patients with CCC was similar to that of miR-146a, there was no correlation. Other reports have demonstrated that miR-146a enhances the angiogenic activity of endothelial cells in hepatocellular carcinoma by promoting the expression of platelet-derived growth factor receptor *α* [12]. In addition, miR-146a induces angiogenesis in human umbilical vein endothelial cells* via* upregulation of FGFBP1 signaling [13].

Of note, there are conflicting reports about roles of miR-146a during angiogenesis. MiR-146a, first identified as an inflammation-related miRNA [14], is upregulated in endothelial cells upon exposure to proinflammatory cytokines [15]. Overexpression of miR-146a, in turn, inhibits adhesion molecules such as ICAM-1, VCAM-1, and E-selectin, and it also inhibits the expression of other genes associated with inflammation [16]. Those adhesion molecules, together with inflammation, can trigger endothelial cells (EC) activation, which is viewed as the first step of angiogenesis [17]. It has been further confirmed that inhibition of VCAM-1 represses angiogenesis in vitro [18], and that inhibition of ICAM-1 suppresses the development of CCC in vivo [19]. Although overexpression of miR-146a blunts endothelial activation, knock-down of miR-146a in vitro has the opposite effect, as does deletion of miR-146a in mice [20]. Therefore, further research is necessary to verify the proposed roles of miR-146a during angiogenesis or the development of CCC.

In addition, our data showed that the difference between prevalence of hypertension in poor CCC and control groups was statistically significant. Many evidences showed that hypertension impaired the angiogenesis or CCC formation [21, 22]. The hypertension-related impairment of angiogenesis was associated with restrain of EC activity and VEGF expression, which are important for angiogenesis [21, 22]. Interestingly, other reports found that patients with coronary artery disease more often developed coronary collateral circulation in the presence of arterial hypertension [23]. In addition, hypertension could cause the change of microRNAs such as MiR-21, miR-122, and miR-637 [24]. The plasma miR-146a might be affected by hypertension, which needed further investigation.

## 5. Conclusions

We found that the level of circulating miR-146a positively correlates with Rentrop grades. Moreover, our ROC analysis established that plasma miR-146a could be a potent independent predictor for the development of poor CCC. As is well-known, current methods to evaluate CCC are limited to invasive procedures, such as of coronary angiography and grading. Although clinical parameters associated with the development of CCC had been identified in other studies, there remained a lack of biomarkers for CCC. Thus, we conclude from our study that the level of circulating miR-146a is of great significance in a clinical setting to provide appropriate information about the development of poor or good CCC in patients with CAD.

## 6. Limitations

In this study, the sample size was small and was assembled from a single center. Therefore, further studies on a larger sample size will be needed to verify the current results. In addition, the Rentrop scoring system was used for collateral grading even though small-caliber microvascular vessels may not be visualized by angiography. Moreover, U6 as reference gene was found a less-stable expression than other microRNAs in plasma or serum sample [25]. For determination of plasma miRNA,* Caenorhabditis elegans* miRNA (celmiR39) is most usually used as exogenous control, and U6 is used as endogenous control [26, 27]. Although it was demonstrated that U6 or cel-miR-39 used as the reference gene for plasma miR-146a yielded a similar result [28], the suitable reference gene was needed to evaluate the circulating miRNAs.

## Figures and Tables

**Table 1:** Basic clinical characteristics.

Characteristics	Good CCC (*n* = 34)	Poor CCC (*n* = 44)	Controls (*n* = 34)
Age (years)	62.13 ± 8.02	56.13 ± 7.53	54.24 ± 7.36
Male/female	16/18	25/19	17/17
Body Mass Index (kg/m^2^)	25.10 ± 3.82	24.84 ± 3.61	24.72 ± 3.72
BUN (mmol/L)	5.98 ± 1.46	5.77 ± 1.51	5.45 ± 1.23
Creatinine (*μ*mol/L)	80.3 ± 12.9	82.5 ± 11.7	81.7 ± 12.2
LDL cholesterol (mmol/L)	2.58 ± 0.95	2.79 ± 1.09	3.24 ± 1.31
Triglycerides (mmol/L)	1.86 ± 1.45	1.47 ± 0.87	1.82 ± 0.57
Total cholesterol (mmol/L)	4.58 ± 1.31	4.81 ± 1.56	4.42 ± 1.33
Hypertension	16	25^*∗*^	11
Diabetes mellitus	10	7	5
Smoking	11	23	15

Data are provided as the mean ± SD. CCC, coronary collateral circulation; BUN, blood urea nitrogen; LDL, low-density lipoprotein; ^*∗*^poor CCC versus control group, *P* < 0.05.
